# Effect of 17β-Estradiol on Growth and Biosynthesis of Microalgae *Scenedesmus quadricauda* (CPCC-158) and Duckweed *Lemna minor* (CPCC-490) Grown in Three Different Media

**DOI:** 10.3390/plants11131669

**Published:** 2022-06-24

**Authors:** Tatiana A. Kozlova, David B. Levin

**Affiliations:** 1 Laboratory of Controlled Photobiosynthesis, Timiryazev Institute of Plant Physiology of RAS, Botanicheskaya 35, 127276 Moscow, Russia; 2 Laboratory of Ecology, Institute of Natural and Technical Systems of RAS, Teatralnaya 8A, 354000 Sochi, Russia; 3 Department of BioSystems Engineering, University of Manitoba, Room E2-370, 75A Chancellor’s Circle, Winnipeg, MB R3T 2N2, Canada; david.levin@umanitoba.ca

**Keywords:** aquaponics, steroid 17β-estradiol, *Scenedesmus quadricauda*, *Lemna* *minor*, fish wastewater, population growth, biomolecule synthesis

## Abstract

As fish farm wastewaters have detectable levels of fish hormones, such as 17β-estradiol (E2), an understanding of the influence of fish steroids on algal (*Scenedesmus quadricauda*) and duckweed (*Lemna* *minor*) physiology is relevant to the potential use of fishery wastewaters for microalgae and plant biomass production. The study was conducted using three types of media: Bold Basal Medium (BBM), natural fishery wastewater (FWW), and reconstituted fishery wastewater (RFWW) with the nutrient composition adjusted to mimic FWW. During the experiment, the media were aerated and changes in the pH and conductivity of the water were closely monitored. E2 promoted the growth of *S. quadricauda* and *L.* *minor,* with significant accumulation of high-value biomolecules at very low steroid concentrations. However, clear differences in growth performance were observed in both test cultures, *S. quadricauda* and *L. minor,* grown in different media, and the most effective hormone concentrations were evidently different for the algae and the plant.

## 1. Introduction

There is a great difference in the ability of different organic compounds to bind to and cross cell membranes under conditions that promote cell growth. Most organic molecules bind strongly to ions dissolved in water, which normally reduces membrane permeability, and thus, the bioavailability of both molecules [1,2]. Consequently, the concentrations of dissolved ions (hardness, conductivity), pH, light quality and quantity, and temperature, are among the major factors that influence the impact of organic compounds on the growth and biosynthesis of microalgae and aquatic plants [3,4,5].

Numerous reports provide the range of residual 17β-estradiol (E2) in surface and ground waters as 0.1 to 200 ng/L E2 in North America and Europe [5,6,7,8,9]. In fish-farming wastewaters, the concentration of E2 is much higher, reaching 100–200 µg/L [10,11,12]. With respect to the fate of estradiol in aqueous environments and aquaponic systems, three major processes must be considered. The first process is the “reverse uptake” of steroid hormones into fish, although the percentage of E2 reabsorbed by fish is small [13], and in the case of water flowing out of fish tanks to microalgae and/or plant production units, this possibility can be ignored. The second consideration is abiotic degradation (e.g., photodegradation) of E2. It has been established that between 2% and 10% of spiked E2 concentrations may be photodegraded in 48 h [14]. The third process is biotic degradation of the steroid by the microbial community (bacteria and/or microalgae) of the system. Most exogenous estrogens undergo biotic degradation, which can be simply described as an oxidative process beginning with hydroxylation that increases the solubility of estrogen, followed by glycosylation and methylation. During this process, E2 is converted to estrone (E1), then to estriol (E3) and hydroxyestrone [15] plus low concentrations of an unknown metabolite [3,5,16]. A recent paper by Liu et al. [5] reported the ratio of extracellular (adsorbed) to intracellular (absorbed) E2, as well as residual E2 in the water, after four days of exposure, which appeared to be dependent on the initial concentration of E2, time, and water chemistry. Duckweed was shown as a more powerful agent for removing estrogens from water when compared to microalgae [3]. However, in this work, the authors report that algae and duckweed equally adsorb only 5% of dissolved estrogen at a given time and that the process of absorption and further biodegradation of estrogens occurs quickly.

Different algae species have been shown to remove and degrade estradiols from aquatic environments via biodegradation or biotransformation processes, rather than simple adsorption and accumulation in the cells [5,17]. The ratio of adsorbed to biodegraded estradiols varies among the studies available in the literature but is often reported more or less as half and half [15]. However, some studies suggest that most of the adsorbed estradiol becomes absorbed and biodegraded [5,18] and, importantly, does not accumulate intracellularly, at least not when the concentrations of estradiol were below 100 ng/L [18].

It has been reported that mammalian sex steroids can be synthesized by plants. About 70–80% of plant species tested were found to synthesize progesterone (including 17, 20β-P) and androgens. Estrogens were found to be synthesized by about 50% of the plant species tested [19,20]. The metabolic pathways of sex steroids in plants are suggested to be very similar to those in animals and humans [21,22,23] and that the same enzymes carry out these reactions in animal cells (for example, 17β-hydroxysteroid dehydrogenase). It is likely also true for microalgae species, but research on mammal steroid synthesis by algae cells is not as well documented.

The waterborne hormone, E2, has been shown to promote plant and microalgae growth and biosynthesis. Increases in the biomass of plant shoots and roots have been observed in *Medicago sativa* L. plants at 5 to 500 ng/L, while 50 µg/L E2 and higher was toxic for this plant [24]. Other studies confirmed the positive effect of E2 on flowering in *Cichorium intybus* and *Arabidopsis thaliana* [25,26], as well as an improvement in the development of reproductive organs in *Salvia splendens* [27]. Thus, the processes targeted by estrogens in plants are primarily the early stages of plant development (shoots and young roots) and the formation of the sexual reproduction organs [19,28,29]. In aquatic plants, and particularly in the members of the family Lemnaceae, E2 was reported to increase chlorophyll and total carotenoid content [30], nucleic acids (DNA and RNA), soluble protein content, and reducing sugars content [31]. Stimulation of *L. minor* flowering by E2 was also observed [32].

The growing interest in microalgae biomass production for biofuels, pharmaceuticals, and the agricultural industry has motivated investigations of the influence of steroids, which could be found in fishery wastewater in nano-concentrations, on algal growth and biosynthesis, although the initial studies on the subject of steroid influences on microalgae were conducted more than 45 years ago [33]. It was demonstrated that E2 increases growth and biomass production, as well as the biosynthesis of chlorophyll, carotenoids, proteins, and sugars in *Ch. vulgaris* cells [34,35,36,37] when concentrations between 0.3 to 3000 ng/L exogenous E2 were applied.

Our particular interest in investigating the effect of steroids, commonly detected in fishery wastewater, on the physiology of potential objects for biomass production is dictated by the objectives of a joint scientific and industrial project between the University of Manitoba and Myera Nu-Agrinomics Group Canada Inc. In our previous study on the influence of the estrogen E2, and the progesterone 17, 20β-P, on the microalgal species, *S. quadricauda*, we observed a positive effect of both steroids on the rate of cell growth, the final cell density, and the biosynthesis and accumulation of chlorophyll-a, total carotenoids, and lipids [38]. In the present study, the general goal was to evaluate the ability of low concentrations of the common fishery wastewater steroid E2 to induct growth and commercially valuable biomolecule accumulation in two aquatic organisms proposed for biomass production. Thus, growth parameters and soluble protein production of both *S. quadricauda* and the duckweed species, *L. minor*, as well as pigment (chlorophyll-a and total carotenoids) accumulation in *S. quadricauda* were investigated. An important subject of the study was the evaluation of the steroid influence on aquatic organisms in three different media. Therefore, our experiments were conducted in three different media—Bolt Basal Medium (BBM), fishery wastewater from a hatchery farm (FWW), and reconstituted fishery wastewater (RFWW)—over an 11-day period. RFWW was designed based on the analyzed chemical parameters of fishery wastewater from the trout farms of Manitoba, MB. Changes in water chemistry (pH and conductivity) were assessed, and the concentrations of the major macro- and micronutrients of FWW were analyzed (Tables S1 and S2).

## 2. Materials and Methods

### 2.1. Strains and Culturing Conditions

The test organisms, *Scenedesmus quadricauda* (CPCC-158) and *Lemna minor* (CPCC-140), were obtained from the Canadian Phycological Culture Centre (CPCC; Waterloo, ON, Canada). *S. quadricauda* was chosen as it is known to grow well on wastewaters, exhibiting high biomass, lipid, and carotenoid production [39,40,41]. *L. minor* was selected as a model plant in this study due to its high growth rate and its ability to accumulate high concentrations of protein, carotenoids, and lipids [42,43,44].

A stock culture of algae was grown aseptically in standard Bold’s Basal Medium (BBM) [45] with some modifications (NH_4_Cl was added to 4.5 ppm) for at least two months prior to the experiment at 23 °C, pH 6.9 ± 0.1, with Plant Grow fluorescent lighting at 4000 lux (120 μmol/m^2^/s) measured with a CalRight CI-1010 digital lux-meter (CalRight Instrument Inc., San Diego, US) in a chamber shaker Innova-44R (New Brunswick Scientific, Enfield, US). A stock culture of duckweed was grown aseptically in standard 30% Hoagland’s medium (30% HG) in the same chamber shaker.

Prior to the experiments with altered ambient conditions (e.g., lighting in the walk-in growth chamber, aeration) all cultures were acclimated to the experimental conditions for a minimum of one month. Before starting the experimental work, the status of the cultures was checked using the health criteria identified by Environment Canada [46,47]. This ensures the quality and relevance of the obtained data. The seeding density of *S. quadricauda* cells in all trials was 6.0 ± 1.0 × 10^4^ cell/mL. The initial density of the *L. minor* culture was ten plants with three fronds on each.

**Figure 1:**
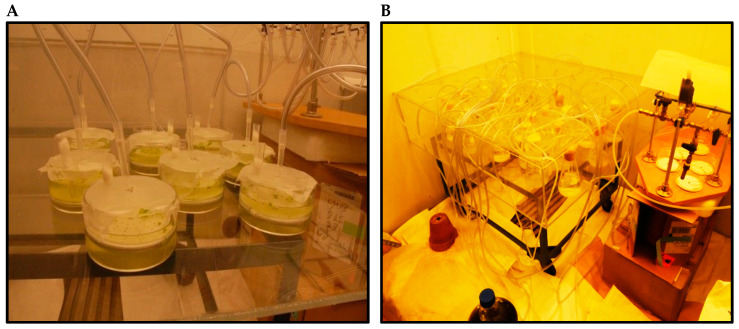
The experimental setup for duckweed in the 300 mL glass bowls (**A**) and microalgae in 250 mL Erlenmeyer flasks (**B**) grown with aeration through plastic discs with 2 mm pores and a flow control manifold (**A**,**B**) in a walk-in chamber. Photo by Kozlova T.A.

All experiments were conducted over an 11-day period. Aeration was applied aseptically to 250 mL Erlenmeyer flasks containing *S. quadricauda* and 300 mL glass bowls containing *L. minor* (Figure 1A,B). The air bubbled through the cultures was dispersed through the plastic discs with 2 mm pores situated at the bottom of the culture bowls (Figure 1A) and Erlenmeyer flasks (Figure 1B). This experiment was conducted in a walk-in growth chamber with controlled lighting (Plant Growing fluorescent Lamp at 4000 lux (120 μmol/m^2^/s) and temperature (23 °C ± 1) (Figure 1A,B).

### 2.2. Water Chemistry

The monitoring of general water chemistry and ambient conditions was performed daily: temperature, pH, and conductivity were measured using an Orion pH-meter 420 (ThermoFisher Scientific Ltd., Waltham, WA, US). The composition of dissolved nutrients (phosphate, nitrate, nitrite, and ammonium ions) and their depletion were monitored using a HACH DR-900 colorimeter (HACH Co., London, ON, Canada). Analysis of water ions (Ca, Mg, K, P, and metals) was conducted by inductively coupled plasma mass spectrometry (ICP-MS) at the Manitoba Chemical Analysis Laboratory (MCAL) of the University of Manitoba. Water samples for ion analysis were acidified with 16N HNO_3_ (1% acidification).

The experimental framework was completed on three different media. The RFWW was designed taking into account both BBM and natural fish wastewater (FWW) physico-chemical parameters. To eliminate the possible influence of pH on *S. quadricauda* and *L. minor* physiology and steroid performance, the pH of all the media tested was adjusted to 7 with 1 N HCl or 1 N NaOH solutions. All pH-adjusted media were equilibrated for 1 day prior to being used in the tests.

### 2.3. The Range of E2 Concentrations

The range of E2 concentrations used in these experiments was based on the previously reported steroid loading in hatchery wastewater, surface waters, and groundwater in North America [48,49] and the reported impact of E2 on vascular plants and algae [15,18]. The range of E2 concentrations tested was 0.25, 0.75, 2.50, 5.0, and 15.0 ng/L; the highest tested concentration is approximately 2 × 10^5^ fold lower than EC50 96 h for microalgae (European Chemicals Agency, ECHA (https://echa.europa.eu/information-on-chemicals; accessed on 14 October 2020) and the United States Environmental Protection Agency, EPA US EcoTox Databases (https://cfpub.epa.gov/ecotox/search; accessed on 24 October 2020) and duckweed species [3,50,51]). The same hormone concentrations were tested at the same time on RFWW, FWW, and BBM. Water-soluble estradiol was purchased from Sigma Aldrich Inc., Oakville, ON, Canada (β-Estradiol Watersoluable, Sigma-Aldrich E4389-100mg Lot # SLBF2466V). E2 stock solutions were prepared on distillate water (based on E2 concentration as 5% of chemical powder). A control-blank (no hormone) was assessed in each trial.

### 2.4. Sampling and Analytical Methods

#### 2.4.1. Algal Cell Counting

Cell density was determined by counting with a Bright-Line haemacytometer (Hausser Scientific Com., Horsham, PA, US) using a Nikon-Eclipse-Tɩ microscope (Nikon Instrument Inc., Melville, NY, US) taking 7 to 10 technical replicates. Cell size was assessed using the NIS-Elements-D3.1 software program of the microscope. The cell’s wellness was evaluated by a criterion of the degree of plasmolysis using the same NIS-Elements-D3.1 software program, where critical plasmolysis was at 50% of protoplasm shrank.

As the seeding density in each trial could not be absolutely equal, the proportional rate of increase between each time point was used to assess cell growth, using the following formula:

N_a_ = (N_t_ − N_o_)/t,

where, N_a_ = the calculated cell density, N_o_ = initial cell density, and N_t_ = cell density on day t.

#### 2.4.2. Assessment of Biomass Production

To determine the total dry mass weight (drw), a 20 mL aliquot of well-shaken culture was taken at the end of the experiment (day 11) from each flask into the centrifuge tubes. The cells were pelleted using a Thermo Scientific^TM^ Sorvall^TM^ RC 6 Plus centrifuge at 4500 rpm (2000× *g*) for 15 min. The supernatant was then discharged and the cells were washed with 0.9% NaCl, pelleted again, dewatered, and oven-dried at 60 °C until the weight was stabilized.

#### 2.4.3. Assessment of Chlorophyll-a and Total Carotenoids Concentrations

To determine concentrations of chlorophyll-a and carotenoids, samples were collected according to the sampling schedule, placed on ice, centrifuged, resuspended in 99.8% methanol (Sigma Aldrich Inc.), and stored in the dark for a minimum of 24 h. Aliquots of each extract were transferred into wells of a 96-well plate and measured for optical density using a microplate spectrophotometer (BioTek Synergy 4-Hybrid). Chlorophyll-a and total carotenoids were calculated using the method for methanol extraction described by [52]. Chlorophyll and carotenoid concentrations per cell (ng/cell) were calculated using the following equation:

X = C/N × 1000,

where, X = pigment concentration per cell in ng/cell, C = pigment concentration in µg/mL of extract, and N = number of cells per mL.

#### 2.4.4. Duckweed Assessment

Growth parameters of *L. minor* were assessed according to the methods EPS 1/RM/37 [47] and ISO/FDIS/20079 [53] described by Environmental Canada. A minor adjustment was accomplished for root growth evaluation by taking into account the method described by Greenberg et al. [54]. Soluble proteins of dry duckweed biomass were assessed with a Bradford assay.

### 2.5. Statistical Analyses

To determine the statistical relevance of the data, all trials were conducted using four to five independent biological replicates (n = 4 or 5) with four to fifteen technical replicates which were then analyzed for significant differences (*p* < 0.05) against the control (zero hormone concentration) using the Analysis of Variance test (ANOVA). The significance of measured differences in cell size and biomass was assessed with the Mann–Whitney Rank Sum Test using SigmaStat software (version 3.5, Systat Software, Inc., Palo Alto, CA, US).

## 3. Results

### 3.1. Water Chemistry

The FWW from a hatchery fish farm was screened for major inorganic and organic compounds, and it was found that concentrations of key macro- and micronutrients in the wastewater were sufficient for algae and duckweed biomass production (See Tables S1 and S2 in the Supplementary Materials). Concentrations of copper (Cu) were found to be above LOEC, which is about 1 to 3 µg/L for Cu [55,56]. Reconstituted fishery wastewater was used to evaluate of influence of macro- and micronutrients in FWW, eliminating the possible co-influence of unknown dissolved inorganic and/or organic compounds in the water derived from an actual fish hatchery farm.

Over the 11 days of experiments with *S. quadricauda,* the pH of the growth media shifted to alkaline conditions, but the extent of the pH change was different for each of the three media tested (see Figure S1A–C in the Supplementary Materials). In both artificial media (BBM and RFFW), pH increases occurred to a lesser degree compared with FWW, and it appears that the E2 presence did not influence pH changes in these media at the tested E2 concentrations. However, a change in pH was observed in the FWW medium at E2 concentrations of 0.25 to 2.5 ng/L. The pH increase was significantly less than that observed in the control cultures (with no steroid present) and cultures with 5 and 15 ng/L E2 (Figure S1A). Interestingly, the pH of the FWW medium was more alkaline at all concentrations of E2 tested, compared with the equivalent E2 concentrations in the two artificial media (Figure S1A–C). The conductivity of the media did not change significantly over the 11 days of the experiment (data are not shown). However, the conductivity of RFWW was about 1.3-fold higher than the conductivity observed in BBM and 2.2-fold higher than the conductivity observed in FWW.

### 3.2. Effect of E2 on S. quadricauda and Duckweed (Lemna minor) Growth and Biomass Production

#### 3.2.1. Growth and Biomass Production in *S. quadricauda* under Different E2 Concentrations

**Figure 2:**
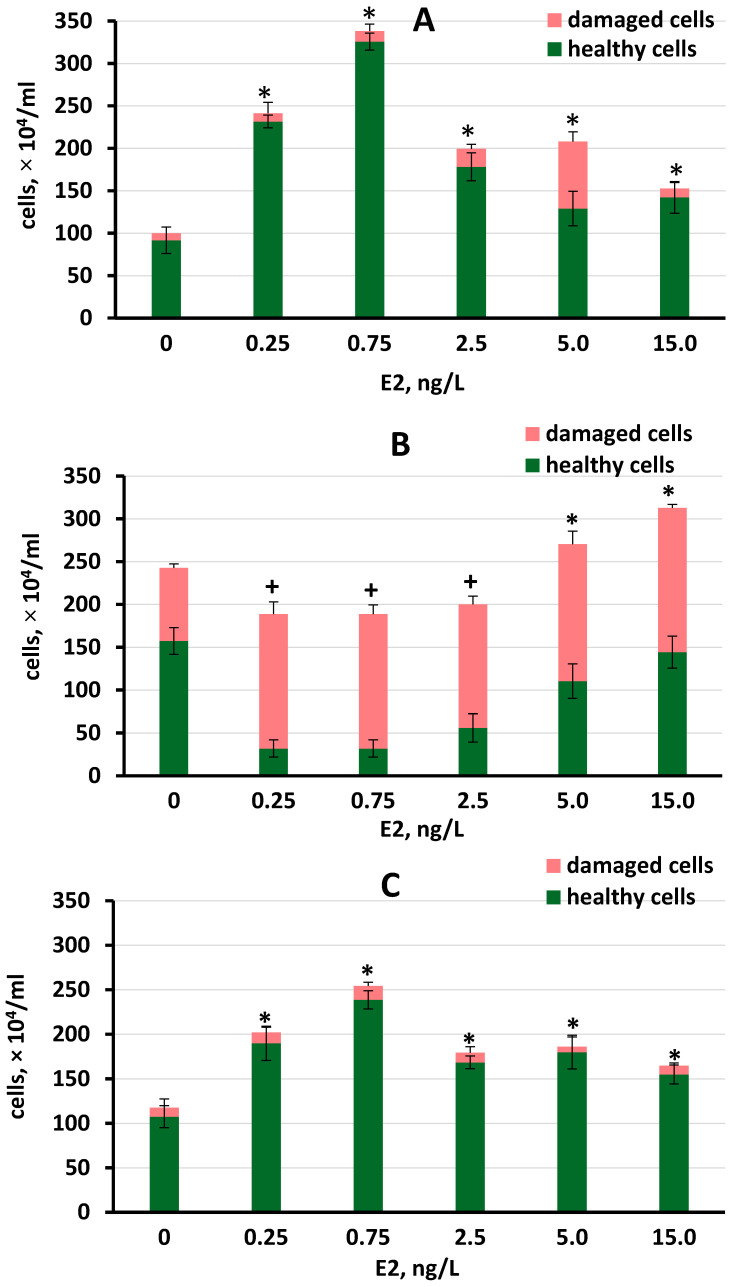
Cell density of *S. quadricauda* cultured in the three media containing E2 concentrations of 0.25 ng/L to 15 ng/L on day 11 of the experiment: (**A**) FFW; (**B**) RFWW; and (**C**) BBM. Statistically significant differences from the controls (*p* < 0.05) are shown for the total cell density, where *—significantly greater than the control; +—significantly lower than the control. Data are shown as the mean ± SD, n = 15. Control cultures did not contain E2, indicated by “0”.

The growth of *S. quadricauda* in the presence of E2 varied with the different types of media (Figure 2A–C). All E2 concentrations tested showed significant stimulation of *S. quadricauda* growth in FWW and BBM. The greatest (3.5-fold) effect was observed in the FFW medium at 0.75 ng/L E2 (Figure 2A). Ranking the growth-stimulating concentrations revealed that 0.75 ng/L > 0.25 ng/L > 2.5 ng/L ≈ 5 ng/L in both FWW and BBM mediums. An E2 concentration of 0.75 ng/L significantly induced greater growth in both FWW and BBM compared to higher tested hormone concentrations.

In RFWW, E2 inhibited the growth of *S. quadricauda* at concentrations between 0.25 and 2.5 ng/L not only compared to the control but also compared to the higher concentrations (*p* < 0.05). In addition, a high percentage of unhealthy cells was observed in all cultures containing E2 (Figure 2B). E2 concentrations of 5 and 15 ng/L increased total cell density, although the percent of healthy cells was lower at these concentrations compared to the control. *S. quadricauda* grown in control (no E2 added) in FWW and BBM at the end of the experiment increased cell density to a similar degree (Figure 2A,C). However, in the RFWW trial, the numbers of healthy, unhealthy, and total cell density were significantly greater in the control (2.0–2.4 times) compared to these numbers in the other two media (Figure 2A–C).

Biomass yields of *S. quadricauda* were significantly higher in FFW and BMM at all E2 concentrations tested compared with the control cultures (Table 1). The greatest increase in biomass production (4.5-fold) was observed in the FWW medium at 0.75 ng/L E2. No increase in biomass production was observed in the RFWW medium. Moreover, concentrations from 0.25 to 2.5 ng/L E2 demonstrated adverse effects on biomass yield on day 11 of the experiment. When comparing biomass production in the controls, the highest value was obtained in RFWW. Biomass yields of *L. minor* were positively affected by most tested E2 concentrations in all three media (the only exception was 15 ng/L E2 in BBM where no effect was observed). The greatest increase in biomass production was in the RFWW medium with a maximum increase of 2.4-fold at 0.75 ng/L despite the fact, that the highest biomass yield in control was in the FWW medium (Table 1).

*S. quadricauda* cell size was affected differently by E2 in all three media. In FWW and RFWW, cell size was not affected, except at the highest tested E2 concentration of 15 ng/L, at which an adverse effect was observed (Table 1). In BBM medium, concentrations of 0.25 to 2.5 ng/L E2 resulted in significantly increased cell size, with no effect being observed at 5 and 15 ng/L. Higher diversity in cell size was noted in the RFWW medium at all E2 concentrations tested (Table 1), although the cell size in the controls prepared on FWW and RFWW media was diverse too. No change in cell size was observed in the control cultures of all three media during the 11 days of the experiment. The ability of E2 to induce growth and biosynthesis of both tested organisms depends on the tested concentration and the parameter, sometimes showing a greater effect at a smaller concentration. (Table 1).

#### 3.2.2. *L. minor* Growth under Different E2 Concentrations in Three Media

**Figure 3:**
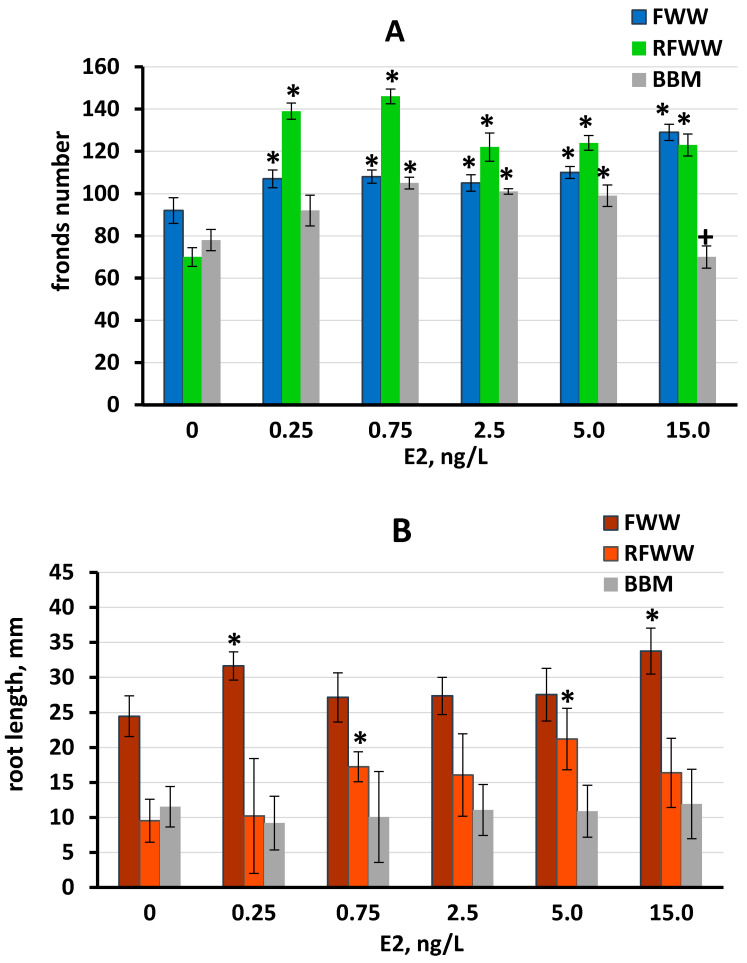
Growth of *L. minor* cultured in the three media containing E2 concentrations of 0.25 ng/L to 15 ng/L, on day 11 of the experiment: (**A**) foliage development in FFW, RFWW, and BBM media; (**B**) root length in FFW, RFWW, and BBM media. Statistically significant differences from the controls (*p* < 0.05) are indicated, where *—significantly greater than the control; +—significantly lower than the control. Data are shown as the mean ± SD, n = 15. Control cultures did not contain E2, indicated by “0”.

The influence of E2 on *L. minor* growth was assessed by measuring the development of the roots and fronds. All E2 concentrations tested significantly stimulated the growth of *L. minor* fronds, in all three media, except in BBM at 0.25 and 15 ng/L E2 (Figure 3A), with the greatest effect observed in RFWW at 0.25 and 0.75 ng/L E2. The influence of E2 on frond development was comparable in FWW and BBM media, while the stimulation of *L. minor* fronds was greatest in the RFWW medium (Figure 3A). Stimulation of *L. minor* root development was observed in FWW at 0.25 and 15 ng/L E2 and in RFWW at 0.75 and 5 ng/L E2. However, no significant influence on root development was observed at any E2 concentration in the BBM trial (Figure 3B). The variation in root length was significant in all three media, although the SD was lower in FFW compared to the other media.

### 3.3. Effect of E2 on Chlorophyll-a and Total Carotenoid Production by S. quadricauda Cells

**Figure 4:**
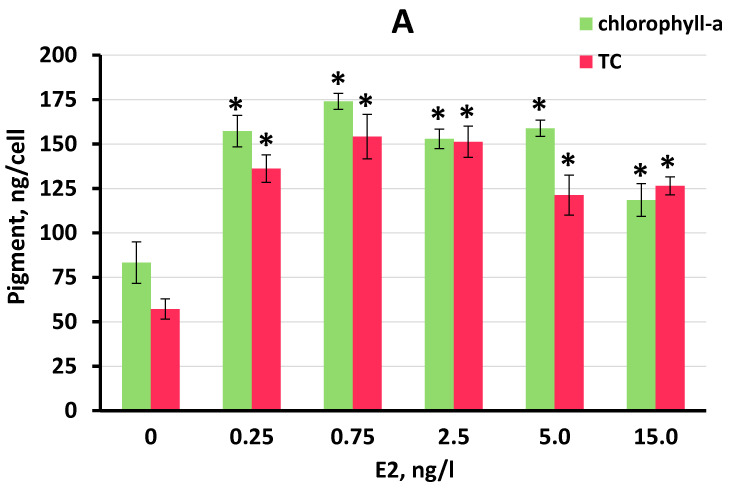

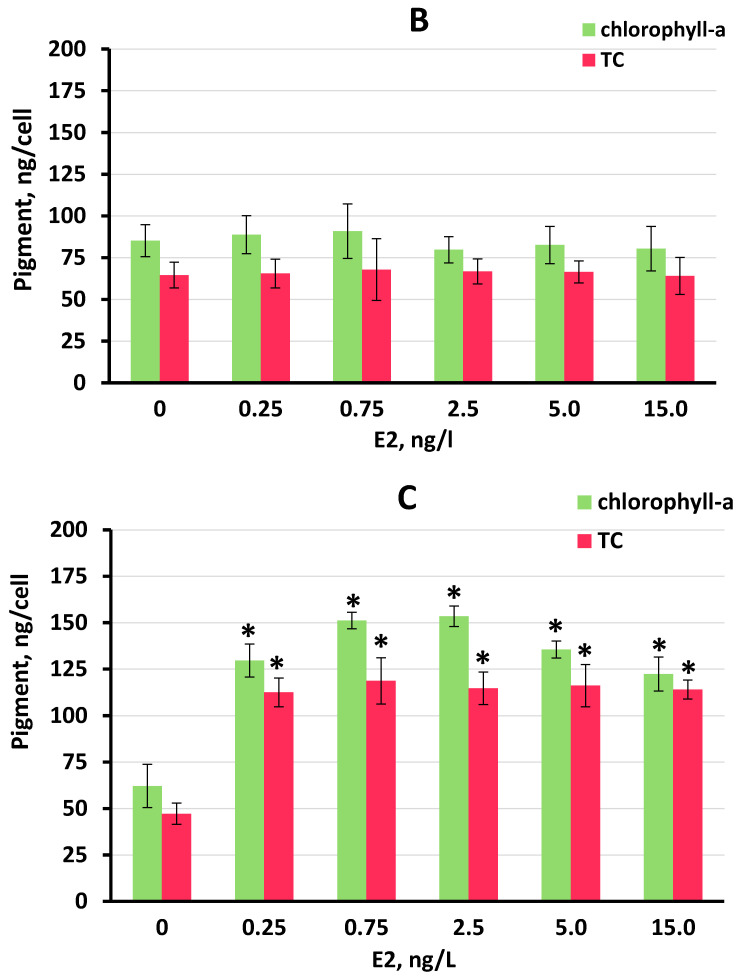
Chlorophyll-a and total carotenoid (TC) content in *S. quadricauda* cells cultured in the three media containing E2 concentrations of 0.25 ng/L to 15 ng/L on day 11 of the experiment: (**A**) FWW; (**B**) RFWW; and (**C**) BBM. Statistically significant differences from the controls (*p* < 0.05) are shown for total cell density, where *—significantly greater than the control. Data are shown as the mean ± SD, n = 6. Control cultures did not contain E2, indicated by “0”.

Significant increases in chlorophyll-a and total carotenoids were observed at all E2 concentrations tested in FWW and BBM (Figure 4A,C). In contrast, no increase in either pigment was observed in RFWW (Figure 4B). Concentrations of both chlorophyll-a and total carotenoids increased in *S. quadricauda* cells to higher levels than in untreated control cells in the FWW medium. A maximum chlorophyll-a concentration of 174.1 ng/cell was obtained in FWW at 0.75 ng/L E2, although at E2 concentrations of 0.25 to 5 ng/L, chlorophyll-a concentrations increased by 1.8- to 2.1-fold compared to the untreated control cultures.

In this trial, the maximum concentration of total carotenoids was nearly equal at 0.75 and 2.5 ng/L E2 (154.2 and 151.7, respectively), with an increase of 2.7-fold over the concentrations of total carotenoids observed in the untreated control cultures (Figure 4A). Ranking the efficacy of stimulation of chlorophyll-a by E2 in FWW, we determined that: 0.75 ng/L > 0.25 ng/L ≥ 5 ng/L ≥ 2.5 ng/L > 15 ng/L. Ranking the efficacy of stimulation of total carotenoids by E2 in FWW, we determined that: 0.75 ng/L ≈ 2.5 ng/L > 0.25 ng/L ≥ 5 ng/L ≥ 15 ng/L.

In BBM medium, maximum values of 153.4 and 151.2 ng/cell of chlorophyll-a were observed at 2.5 and 0.75 ng/L E2, respectively (Figure 4C). The accumulation of chlorophyll-a was stimulated to a lesser extent at 0.25, 5, and 15 ng/L E2. Total carotenoid accumulation in *S. quadricauda* cells was stimulated 2.4- to 2.5-fold at all E2 concentrations tested in BBM medium (Figure 4C). Ranking the efficacy of stimulation of chlorophyll-a in BBM medium, we found that 2.5 ng/L ≈ 0.75 ng/L > 5 ng/L ≥ 2.5 ng/L ≥ 15 ng/L. Ranking the efficacy of stimulation of total carotenoids in BBM medium, we found that 0.75 ng/L ≥ 2.5 g/L ≈ 5 ng/L ≈ 15 ng/L ≥ 0.25 ng/L. Between the tested E2 concentrations, the stimulatory effect was not significantly different, except for the highest tested E2 concentration (15 ng/L) in FWW where both pigments were reduced compared to other E2 concentrations.

### 3.4. Soluble Protein Production by L. minor and S. quadricauda

Accumulation of water-soluble protein in *L. minor* and *S. quadricauda* biomass was different when the test organisms were grown in three media containing the range concentrations of E2, compared to untreated control cultures (Table 2). In the untreated control cultures, both *L. minor* and *S. quadricauda* cultured in the FWW medium produced the highest concentration of soluble proteins (36.12 and 30.11 mg/g frw, respectively), compared with the untreated controls cultured in RFWW and BBM. In the presence of E2, elevated protein concentrations (compared with the untreated control cultures) were observed in *L. minor* and *S. quadricauda* cultured in FWW at concentrations of E2 from 0.25 to 15 ng/L, although the greatest stimulation of protein accumulation in both cultures was at 2.5 ng/L. In RFWW, elevated protein concentrations were observed in cultures containing 0.25, 0.75, and 2.5 ng/L E2 for *L. minor* and in *S. quadricauda* cells at any tested concentration (compared with the untreated control cultures). In BBM medium, protein accumulation was different for the two tested cultures. For *L. minor* tissue, elevated concentrations of protein compared with the untreated control cultures were only detected in a medium containing 15 ng/L. *S. quadricauda* cells reacted stronger in the presence of E2. The concentrations from 0.75 to 15 ng/L E2 were effective in elevating the algae protein content (Table 2). Overall, E2 was more effective in the induction of soluble protein accumulation in the algae compared to duckweed. Between the tested E2 concentrations, the greatest effect (2.0 fold induction) demonstrated 15 ng/L E2 in BBM in *S. quadricauda*, while the weakest difference in induction was observed in *L. minor* grown in BBM (Table 2).

## 4. Discussion

The range of E2 concentrations examined in this experiment was consistent with the concentrations typically found in Canadian surface and ground waters, and fish-farming wastewaters [57,58,59]. Moreover, a reduced range of steroid concentrations was chosen for investigation, considering two additional important points: (a) toxicity of the hormones to plants and algae; and (b) in an attempt to avoid the possibility of hormone accumulation in the algal cells. Toxicity of E2 to microalgae is relatively low, normally more than 3.2 mg/L 96 h EC50 and [5], but is from 0.2 mg/L in some reports (ECOSAR v 2.2) depending on the species and water chemistry. NOEC of E2 is in the range of 10 to 100 µg/L with the lower toxicity of natural estradiol (EPA US EcoTox Database).

The chemical compound E2 that we used in this study contains cyclodextrin (β-CD) as a concomitant that makes E2 water-soluble. This may raise the question of the co-influence of β-CD on the effect of E2 or the bioavailability of steroids. However, according to the information on β-CD provided in the Material Safety Data Sheet (MSDS, Sigma-Aldrich) and ECHA and the EPA US EcoTox Databases, EC50 and even NOEC, for β-CD, demonstrated very low toxicity or no impact of the compound on the physiology of microalgae species and other aquatic organisms (LC50 > 100 mg/L and up to 7500 mg/L; NOEC 30–40 mg/L). Thus, the highest expected β-CD concentration in our experiment is 285 ng/L, which is about 10^5^ lower than NOEC concentrations for green algae species. One would not expect any co-influence of β-CD in such concentrations, especially in conditions of intensive stirring during the preparation of the stock solutions and subsequent aeration of the test media. The matter of solvent concentration was clearly demonstrated in our previous study on phytohormones’ influence on microalgae physiology [60]. The influence of dissolved compounds on each other as well as on a test organism is highly dependent on the concentration of each compound in the solution and lesser on the ratio of two concomitant substances (in our case E2 and β-CD) [50,61]. Numerous studies, which used β-Estradiol water-soluble (Sigma-Aldrich E4389) did not conduct additional tests on the influence of β-CD, when much higher E2 concentrations, compared to our study (the difference is about 1 × 10^3^ 7 × 10^5^ fold), were examined [62,63,64,65]. However, an additional test on β-CD influence was not conducted in these studies, likely due to the significantly lower concentration used, compared to EC50 48h β-CD (ECHA and EPA US EcoTox Databases).

E2 is one of the frequently detected hormones in fishery wastewaters, which is why it may be used as a beneficial component of growth media in combined fish-algae-duckweed integrated production systems. In our previous study [38], E2 demonstrated a powerful ability to simultaneously induce the growth and biosynthesis of valuable bio-molecules in *S. quadricauda* cells. The current study was conducted to clarify whether the influence of E2 on the algae could be affected by physicochemical differences in three different growth media, including RFWW, which mimics the concentrations of macro- and micronutrients in real fish wastewaters (FWW). This mimicry was made for the possible replacement of FWW for further biomass production in pilot-scale facilities. Moreover, this study compares the effect of E2 on *S. quadricauda* physiology, with the steroid influence on *L. minor*, when these two aquatic organisms were grown under the same conditions.

Although ambient conditions in our previous study had some dissimilarities from this experimental work (the light intensity was 20% higher and constant aeration of the growth mediums was not provided during the first experiment), the results of E2 influence on *S. quadricauda* growth parameters and biosynthesis of pigments were similar, comparing the values on days 10 and 11 of the experiments conducted in BBM medium.

However, clear dissimilarities in the algae population growth and biosynthetic activity were observed in three media tested. Remarkably, the dissimilarities in algae performance were found between the controls of three trials as well as between the two test cultures containing equal concentrations of E2. Furthermore, the pattern of dissimilarity between the controls varied with the type of medium used. For instance, cell density was about two times higher in untreated control cultures in RFWW media compared to the control cultures with BBM and FWW media. However, when E2 was present in the cultures, the effect was the opposite: in RFWW, cell density and biomass yield of *S. quadricauda* were negatively affected by the three lowest E2 concentrations (0.25, 0.75, and 2.5 ng/L), while in FWW and BBM, these concentrations stimulated an increase in cell density and biomass yield.

The RFWW medium was prepared according to the results of chemical analyses of actual FWW from two trout farms. The macro- and micronutrients of FWW were incorporated into the design. The main metals, which could cause toxicity of FWW, were also analyzed and were found to be under LOEC for each metal [66,67], except Cu, which was slightly higher (Table S2). Why E2 negatively affected cell growth in the RFWW and why RFWW compromised cell health needs further investigation. A possible reason may be related to the buffering capacity of the medium, where sodium and potassium carbonates were used to stabilize the pH of RFWW. This buffering strategy decreased the shifting of the pH to alkaline conditions and increased the dissolved carbon value, which could directly affect the binding capacity of E2, and/or membrane permeability [3]. It is also possible that the period of time for cells’ acclimatization to the RFWW media was insufficient for the algae to stabilize its physiology prior to the test. When considering the effect of water chemistry on chlorophyll-a and carotenoids, it may be that the stimulatory effect of E2 observed in the FWW and BBM media was abolished in the RFWW medium.

In contrast to RFWW, the positive effect of E2 on *S. quadricauda* growth and biosynthesis in natural FWW was the most pronounced. The effect of water chemistry on the promoting efficiency of an induction factor (such as E2) has been highlighted previously by research conducted on aquatic plants [68,69,70] and microalgal species [71,72,73]. It was demonstrated that a change in water chemistry could activate more than one defense pathway at the same time in algae cells. The combination of these pathways and dominance of one of them depends on the factor altered, whether it is a media chemistry parameter or an additional stressor, such as a hormone, or both [71,74].

Remarkably, the influence of RFWW on *L. minor* growth was opposite to its influence on *S. quadricauda* growth. The growth of foliage, root development, and protein accumulation in the duckweed was stimulated by greater E2 in the RFWW medium, compared with the effect of E2 in the other two media, although FWW was a significantly better medium for *L. minor* growth when E2 was not present. In addition, it is not clear if the greater effect of E2 on protein content in RFWW was due to the altered water chemistry. Soluble protein concentrations in *S. quadricauda* cells in all the tested mediums were significantly lower than in *L. minor* tissue. However, E2 was more effective in the induction of soluble protein accumulation in the algae compared to duckweed. Coupled with the fact that induction of cell growth and population density by E2 was stronger for *S. quadricauda* compared to the effect of E2 on *L. minor* foliage growth (maximum biomass increase as 4.5-fold and 2.4-fold induction, respectively), we can conclude that E2 is a more effective inductor for *S. quadricauda* than for *L. minor* at the E2 concentrations and experimental conditions tested.

Our results on the influence of E2 on the soluble protein accumulation by *L. minor* are consistent with research conducted on another duckweed species, *Wolffia arrhizal* [31]. However, we observed the maximum effect at much lower E2 concentrations in both RFWW (0.25 and 0.75 ng/L E2) and FWW (between 0.75 and 15 ng/L E2) media, compared to the effect on *W. arrhizal*, where the concentrations of 272, 2720, and 27.2 µg/L E2 (in that order) were most stimulatory. In the study by Szamrej and Czerpak [30,31], soluble proteins were induced to a greater degree (1.8-fold increase) compared to maximum induction in our experiment (1.3-fold increase). The species-specific differences (e.g., *W. arrhizal* does not have roots) and growth media chemistry are likely responsible for the difference in results.

Lemnaceae species are broadly used for wastewater treatment, including domestic and pharmaceutical effluences, which are the prime resources of animal steroids in surface water. Some previous research indicates that duckweed species are one of the best agents for removing hormones from water [3,75,76]. The low E2 waterborne concentrations were likely inactivated and biotransformed by *L. minor* shortly after the exposure. E2 and other animal steroids have also been shown to induce biosynthesis of valuable molecules in Lemnaceae plants, when applied in relatively low concentrations in the range of 10^−5^ to 10^−7^ M. For example, corticosteroids significantly stimulated the accumulation of pigments (chlorophylls and carotenoids), nucleic acids, soluble proteins, and sugar biosynthesis in *W. arrhizal* tissues, although the extent of stimulation was less than the effect of E2 [30,31]. To the best of our knowledge, no study has measured duckweed growth performance in the presence of known concentrations of waterborne steroid hormones. The effect of *L. minor* on buffering and stabilizing the pH of the growth media has also not previously been reported. However, efforts were made for the development of a mathematical model for a better understanding of the influence of growth medium alteration on Lemnaceae performance in vitro and in situ [77,78,79].

Our results on the influence of E2 on *S. quadricauda* growth and biomass production (cell size and density) and the accumulation of pigments (chlorophyll-a and total carotenoids) in BBM medium over 11 days were similar to that obtained in the previous 20-day experiment [38]. However, the stimulatory effects in this study were more pronounced than the effects observed on days 10 to 15 in the previous experiment. The better results are likely due to the constant aeration condition applied in this (11-day) experiment.

## 5. Conclusions

We have compared the influence of an animal steroid, E2 on the growth and physiology of two primary producers: the microalga *S. quadricauda* and the aquatic plant *L. minor*. The results of this study may be organized into two groups. First, it was confirmed that E2 in nano-concentrations has a strong positive effect on the cell growth (cell size and final cell density) and biosynthesis of valuable molecules of *S. quadricauda*, as was previously demonstrated in a 20-day non-aerated experiment. Moreover, we have demonstrated that E2 is capable of increasing foliage growth, root growth, and protein biosynthesis in *L. minor*, although root development was stimulated by the presence of E2 to a lesser extent. Importantly, E2 exhibited a stronger stimulatory effect on *S. quadricauda* compared to *L. minor* when population growth and protein content were assessed. Second, we have compared the influence of three growth media (FWW, RFWW, and BBM) on the two test organisms, both in the absence and presence of E2. The results of this comparison clearly show the importance of the physicochemical parameters of the growth medium on the physiology and metabolism of aquatic organisms, as well as on the extent to which E2 affected the physiology of *S. quadricauda* and *L. minor.* Our results highlight the need for further investigations of the relationships between the physicochemical parameters of growth media, the particular organism to be cultured, and any additional stressor or chemical inducer that may be applied to stimulate biomass production and biosynthesis of the molecules of interest.

## Figures and Tables

**Table 1:** Changes in growth parameters of *Scenedesmus quadricauda* and *Lemna minor* when cultured in the three growth media, on day 11 of the experiment (the day on which the experiment was terminated).

	*S. quadricauda*			*L. minor*
E2 Concentration, (ng/L)	Cell Size (µm)	Biomass (g drw/L)	Cell Density (×10^4^/mL)	Biomass (g drw/L)
**FWW trial**				
Control	44.77 ± 3.26	0.40 ± 0.07	100.07 ± 5.23	3.40 ± 0.05
0.25	46.65 ± 4.73 ^a^	1.15 ± 0.11 *^a^	241.43 ± 3.87 *^a^	5.05 ± 0.09 *^a^
0.75	46.87 ± 2.31 ^a^	1.79 ± 0.05 *^b^	338.34 ± 3.18 *^b^	4.94 ± 0.06 *^a^
2.5	42.05 ± 1.45 ^a^	0.88 ± 0.10 *^c^	199.61 ± 4.93 *^c^	4.86 ± 0.08 *^a^
5	37.23 ± 1.85 ^b^	0.86 ± 0.14 *^c^	208.12 ± 8.89 *^d^	4.89 ± 0.09 *^a^
15	31.65 ± 2.22 ^+c^	0.78 ± 0.08 *^c^	152.63 ± 5.47 *^e^	5.59 ± 0.08 *^b^
**RFWW trial**				
Control	43.77 ± 4.25	0.69 ± 0.09	242.67 ± 4.45	2.49 ± 0.07
0.25	47.14 ± 3.43 ^a^	0.44 ± 0.05 ^+a^	188.81 ± 3.22 ^+a^	5.45 ± 0.07 *^a^
0.75	48.25 ± 2.62 ^a^	0.46 ± 0.04 ^+a^	191.52 ± 3.42 ^+a^	5.98 ± 0.08 *^b^
2.5	39.84 ± 5.66 ^a^	0.41 ± 0.07 ^+a^	200.21 ± 5.25 ^+ab^	4.71 ± 0.09 *^c^
5	35.92 ± 4.32 ^ab^	0.72 ± 0.11 ^b^	270.42 ± 8.61 *^c^	4.86 ± 0.07 *^c^
15	26.52 ± 3.65 ^+c^	0.76 ± 0.06 ^b^	312.71 ± 6.05 *^d^	4.66 ± 0.04 *^c^
**BBM trial**				
Control	44.76 ± 0.92	0.44 ± 0.11	128.51 ± 10.66	2.68 ± 0.07
0.25	52.67 ± 1.01 *^a^	0.85 ± 0.09 *^a^	202.12 ± 12.54 *^a^	3.35 ± 0.08 *^a^
0.75	56.72 ± 1.25 *^b^	1.24 ± 0.04 *^b^	254.24 ± 8.42 *^b^	4.14 ± 0.06 *^b^
2.5	46.74 ± 0.63 *^c^	0.79 ± 0.08 *^a^	179.13 ± 5.57 *^c^	3.89 ± 0.09 *^b^
5	46.84 ± 1.05 ^c^	0.81 ± 0.08 *^a^	186.02 ± 14.05 *^ac^	3.84 ± 0.06 *^bc^
15	42.95 ± 1.48 ^d^	0.75± 0.06 *^a^	164.64 ± 7.33 *^ac^	2.95± 0.09 ^d^

*—significantly greater than control (*p* < 0.05); **^+^**—significantly lower than control (*p* < 0.05). Within a trial, and for each column, values labeled with the same letter are not significantly different from each other (*p* > 0.05) and values labeled with two letters indicate a significant difference from other letters, but no statistical difference from other equal letters. Data are shown as the mean ± SD (for cell size n = 15; for biomass n = 4; for cell density n = 12).

**Table 2:** Protein (mg/g frw) accumulation in *L. minor* grown in three different media on the day of the test termination (day 11).

E2, ng/L	Control	0.25	0.75	2.5	5	15
*Lemna minor*						
FWW	36.12 ± 1.06	34.77 ± 0.76 ^a^	38.95 ± 0.48 *^b^	40.41 ± 0.57 *^b^	38.92 ± 0.91 *^b^	39.68 ± 0.66 *^b^
RFWW	31.73 ± 0.72	40.74 ± 1.21 *^a^	41.12 ± 0.43 *^a^	36.02 ± 0.79 *^b^	32.82 ± 1.04 ^c^	32.51 ± 0.72 ^c^
BBM	24.75 ± 0.66	25.34 ± 0.26 ^a^	25.28 ± 1.01 ^a^	26.25 ± 0.76 ^a^	25.78 ± 0.86 ^a^	27.78 ± 0.58 *^b^
*Scenedesmus quadricauda*						
FWW	30.11 ± 0.56	37.22 ± 1.03 *^a^	43.35 ± 0.19 *^b^	46.76 ± 0.63 *^c^	44.52 ± 1.52 *^bc^	40.14 ± 1.06 *^d^
RFWW	25.88 ± 0.69	29.94 ± 0.21 *^a^	36.02 ± 0.48 *^b^	39.93 ± 0.55 *^c^	43.76 ± 1.04 *^d^	29.57 ± 1.48 *^a^
BBM	20.38 ± 0.21	21.84 ± 0.75 ^a^	31.08 ± 0.61 *^b^	36.25 ± 0.65 *^c^	38.78 ± 0.34 *^d^	41.88 ± 0.71 *^e^

*—significantly greater than control (*p* < 0.05). Within a trial, and for each row, values labeled with the same letter are not significantly different from each other (*p* > 0.05), and values labeled with two letters indicate significant difference from other letters, but no statistical difference from other equal letters. Data are shown as the mean ± SD, n = 6.

## Data Availability

Data are contained within the article and Supplementary Materials. Raw data were generated at the Department of Biosystems Engineering, University of Manitoba and are available from the corresponding author (initials) upon request.

## Supplementary Materials

The following supporting information can be downloaded at: https://www.mdpi.com/article/10.3390/plants11131669/s1. Table S1: Macronutrients and buffering capacity of standard synthetic Bold’s Basal Medium (BBM), 30% Hoagland’s medium (30% HG), natural fishery wastewater (FWW), and reconstituted fishery wastewater (RFWW) used in the experiment, ppm; Table S2: Micronutrients in standard synthetic Bold’s Basal Medium (BBM), 30% Hoagland’s medium (30% HG), natural fishery wastewater (FWW), and reconstituted fishery wastewater (RFWW) used in the experiment, ppm.; Figure S1: pH of three culture media on day 11 of the experiment. Micronutrients in standard synthetic Bold’s Basal Medium (BBM), 30% Hoagland’s medium (30% HG), natural fishery wastewater (FWW), and reconstituted fishery wastewater (RFWW) used in the experiment, ppm.
