# Supplementary material for: Effect of 17β-Estradiol on Growth and Biosynthesis of Microalgae Scenedesmus quadricauda (CPCC-158) and Duckweed Lemna minor (CPCC-490) Grown in Three Different Media
Source: Plants (Basel). 2022 Jun 24;11(13):1669. doi: 10.3390/plants11131669 (PMC9269517; doi:10.3390/plants11131669)
Supplement: Supplementary file 1 [file plants-11-01669-s001.zip › plants-1720697-supplementary.pdf]

Supplementary Materials

Table S1. Macronutrients and buffering capacity of standard synthetic Bold's Basal Medium (BBM), 30% Hoagland's medium (30% HG), natural fishery wastewater (FWW), and reconstituted fishery wastewater (RFWW) used in the experiment, ppm.

	**DOC**	**TC**	**TDS**	**TN**	**NO_3**	**NO_2**	**NH_4-N**	**PO_4-P**	**TDP**	**pH**	**Alkalinity (as $CaCO_3$)**	**Conductivity (mS/cm)**
**BBM**	<0.5	<1	635	41	182	0	1.5	50	50	6.8	354	720
**30%HG**	70	60	25	74	0.25	1.5	<0.005	<0.005	0	0	0	0
**FWW**	19.4	60.4	585.5	20.5	175	0.2	0.7	0.9	30	7.75	363.7	555
**RFWW**	<0.5	50	600	40	180	0	1	50	50	7	350	750

DOC, Dissolve Organic Carbon; TC, Total Carbon ($KHCO_3$; $NaHCO_3$); TDS, Total Dissolved Solids; TN, Total Nitrogen; TDP, Total Dissolved Phosphorus.

Table S2. Micronutrients in standard synthetic Bold's Basal Medium (BBM), 30% Hoagland's medium (30% HG), natural fishery wastewater (FWW), and reconstituted fishery wastewater (RFWW) used in the experiment, ppm.

**Ions**	**Ca**	**Mg**	**K**	**Na**	**B**	**Fe**	**Cu**	**Zn**	**Al**	**Ni**	**Pb**	**Cd**
**BBM**	68	25	62	68	0.5	1.5	0.005	0.005	0	0	0	0
**30%HG**	70	60	25	74	0.25	1.5	<0.005	<0.005	0	0	0	0
**FWW**	73.5	60	16	75.6	0.3	1.06	0.04*	0.007	0.04	0	0.001	0.005
**RFWW**	70	40	52	68	0.5	1.5	0.005	0.005	0	0	0	0

* - above LOEC (Lowest Observed Effect Concentration) for algae species [66, 67].

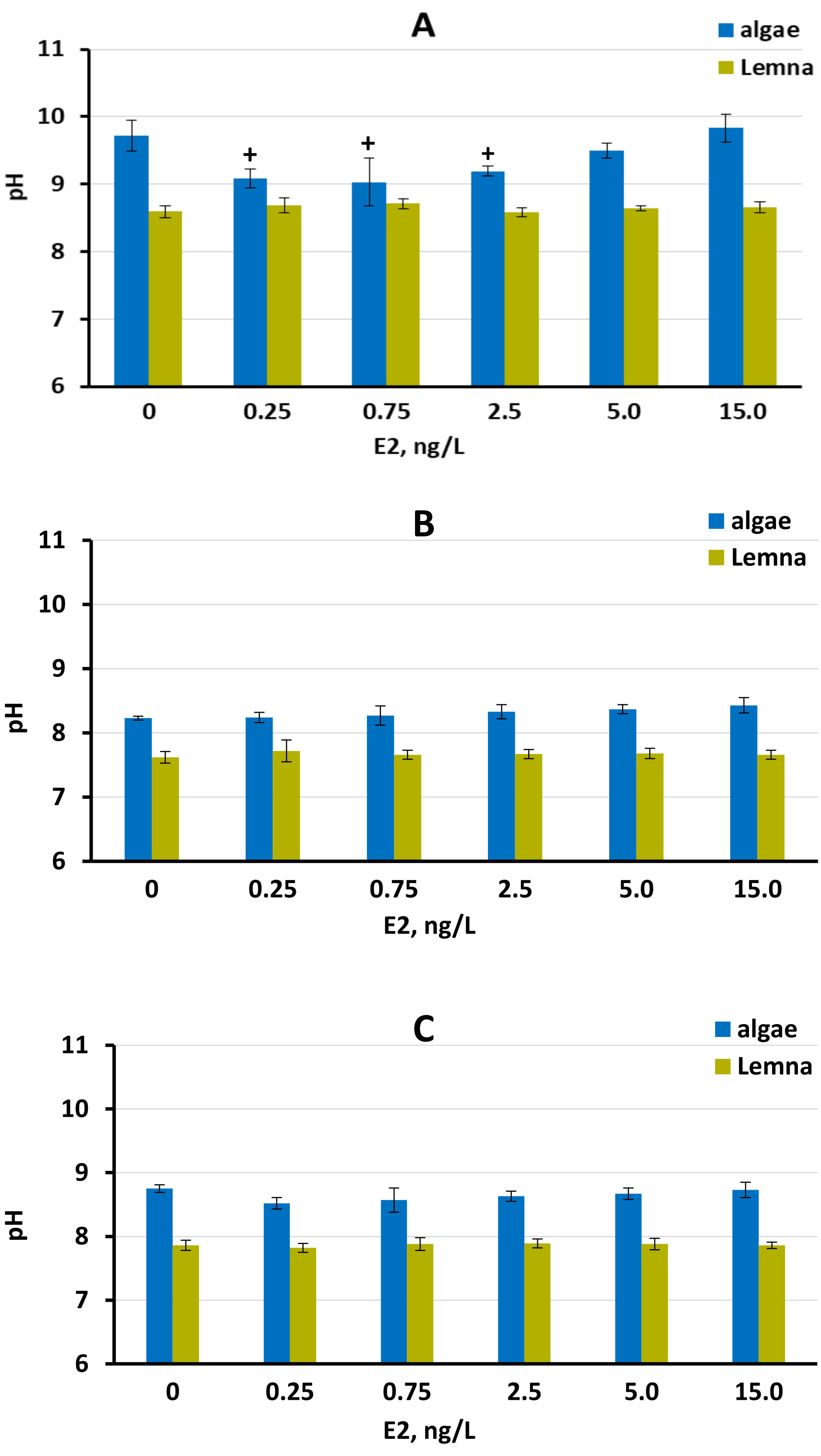
A
B
C
algae
Lemna
pH
11
10
9
8
7
6
0
0.25
0.75
2.5
5.0
15.0
E2, ng/L
+

Figure S1. pH of three culture media on day 11 of the experiment: (**A**) FFW; (**B**) RFWW; (**C**) BBM. Statistically significant difference from the controls ($p < 0.05$) are indicated, where * - significantly greater than the control and + - significantly lower than the control. Data shown as the mean ± SD, n = 4. Control cultures did not contain E2, indicated by “0”.
